# Evaluation of Serum HIF-1α as a Hypoxia-Related Biomarker in Patients with Malignant Salivary Gland Neoplasms

**DOI:** 10.3390/biomedicines14071611

**Published:** 2026-07-17

**Authors:** Wojciech Domka, Maciej Misiołek, Angelika Myśliwiec, Tomasz Kubrak, Agnieszka Przygórzewska, Dorota Bartusik-Aebisher, David Aebisher

**Affiliations:** 1 Department of Otolaryngology, Faculty of Medicine, Collegium Medicum, University of Rzeszów, 35-959 Rzeszów, Poland; 2 Department of Otorhinolaryngology and Oncological Laryngology in Zabrze, Medical University of Silesia, 40-055 Katowice, Poland; maciej.misiolek@sum.edu.pl; 3 Department of Biochemistry and General Chemistry, Faculty of Medicine, Collegium Medicum, University of Rzeszów, 35-959 Rzeszów, Poland; amysliwiec@ur.edu.pl (A.M.); tkubrak@ur.edu.pl (T.K.); dbartusikaebisher@ur.edu.pl (D.B.-A.); 4 Doctoral School of University of Rzeszów, Faculty of Medicine, Collegium Medicum, University of Rzeszów, 35-959 Rzeszów, Poland; ap117623@stud.ur.edu.pl; 5 Department of Photomedicine and Physical Chemistry, Faculty of Medicine, University of Rzeszów, 35-959 Rzeszów, Poland

**Keywords:** HIF-1α, malignant salivary gland neoplasms, hypoxia, biomarker, ELISA

## Abstract

**Background/Objectives:** Hypoxia-inducible factor 1-alpha (HIF-1α) is a transcription factor that escapes proteasomal degradation under hypoxic conditions, translocates to the nucleus, and activates genes involved in anaerobic glycolysis (e.g., GLUT1 and LDH-A) and vascular endothelial growth factor (VEGF) expression. Through its role in tumor progression, angiogenesis, and metabolic reprogramming, elevated HIF-1α levels have been reported in various malignancies; however, its serum concentration in malignant salivary gland neoplasms remains unexplored. This study aimed to assess serum HIF-1α levels in patients with salivary gland malignancies. **Methods:** Serum samples were collected from 30 patients diagnosed with malignant salivary gland neoplasms. HIF-1α concentration was determined using an enzyme-linked immunosorbent assay (ELISA). **Results:** The mean serum HIF-1α concentration was 89.20 ± 44.56 pg/mL. Exploratory analyses demonstrated higher HIF-1α levels in stage III tumors compared with stage II tumors and in high-grade tumors compared with lower-grade lesions. **Conclusions:** This is the first study to quantify serum HIF-1α levels in patients with malignant salivary gland neoplasms. The findings suggest that while HIF-1α may have potential as a biomarker, its potential as a biomarker of tumor aggressiveness or of malignant salivary gland neoplasms is limited due to high interindividual variability. Further studies with larger cohorts and standardized methodologies are necessary to establish reference values and clarify the clinical significance of HIF-1α in salivary gland malignancies.

## 1. Introduction

Salivary glands are divided into larger glands (parotid, submandibular and sublingual) and smaller glands [1,2]. The parotid gland is a serous gland, the submandibular gland produces mainly serous secretions, and the sublingual gland shows a predominance of mucous secretions. Smaller salivary glands produce a mixed serous–mucous fluid [3,4]. Due to the embryologic, physiologic and structural complexity of salivary glands, primary neoplasms of this group are characterized by high phenotypic variation and unpredictable clinical course [5,6]. To date, nearly 40 histological subtypes of these tumors have been distinguished [7].

Malignant salivary gland neoplasms are a rare and highly heterogeneous group of cancers [8,9]. Classified as head and neck cancer (HNC), they account for about 6% of HNC cases according to American Head and Neck Society estimates [10]. Data from the WHO Global Cancer Observatory shows that there were 53,583 new cases of salivary gland cancer worldwide in 2020, with an incidence rate of 0.59 and a mortality rate of 0.23 per 100,000 people per year [11]. There has been a gradual increase in their incidence over the past four decades [10]. Although malignant salivary gland neoplasms can occur in any age group, two-thirds of cases are diagnosed in people older than 55, and the average age of diagnosis is 64 [12]. The five-year relative survival rate depends on the stage of the disease: for stage I, it is 96%; for stage II, it is 77%; for stage III, it is 73%; and for stage IV, it drops to 37% [12,13]. Most malignant salivary gland neoplasms originate in the parotid glands, and to a lesser extent in the sublingual and submandibular glands. Tumors of the minor salivary glands account for less than 15% of all tumors in this group [14,15], most of which are located in the oral cavity, primarily in the hard palate [16,17,18]. Tumors of the larger glands are benign in 70–80% of cases, while more than 80% of tumors originating in the smaller salivary glands are malignant [19,20]. The most common histologic type is pleomorphic adenoma, a benign neoplasm with the potential for recurrence and malignant transformation [21].

Diagnosis of malignant salivary gland neoplasms is based primarily on hematoxylin and eosin (H&E) staining, which remains the gold standard for pathological evaluation. Complementary immunohistochemistry is used, which can provide additional diagnostic information in certain cases [22].

Various therapeutic strategies have been used in recent years, including surgery, radiation therapy and chemotherapy [23]. For benign tumors, local excision or superficial parotidectomy is recommended. Malignant tumors require more extensive procedures, such as total parotidectomy, which carries the risk of facial nerve paralysis [24,25]. In patients with high-grade malignancies and locally advanced lesions of the major salivary glands, complementary radiotherapy has shown a survival benefit over surgical treatment alone [26]. Chemotherapy remains the mainstay of systemic therapy in cases of locally recurrent and/or metastatic disease [27,28].

Despite continuous efforts to identify new approaches to improve treatment and diagnosis, progress over the past three decades has been unsatisfactory. Therefore, it is still necessary to find new, effective biomarkers as tools for the diagnosis and prognosis of malignant salivary gland neoplasms [29].

Oxygen plays a key role in many pathophysiological processes [30]. Hypoxia, defined as a reduction in oxygen availability to cells, tissues or the body, can lead to changes in gene transcription and post-translational modifications of proteins, thereby affecting cellular metabolism [31,32]. Thus, adaptive responses to changes in oxygen availability are essential for maintaining cellular homeostasis and organismal survival [33].

Hypoxia-inducible factors (HIFs) are key regulators of oxygen homeostasis, playing an important role in the development, physiology and pathogenesis of numerous diseases [31,32]. HIFs are a family of transcription factors [34], comprising three major isoforms: HIF-1α, HIF-2α and HIF-3α. Each forms heterodimers with a constitutively active HIF-1β subunit that is independent of oxygen levels [35]. HIF-1 was originally identified as a regulator of erythropoietin (EPO) expression [36,37], but is now recognized as a master regulator of the adaptive response to hypoxia, inducing the expression of hundreds of genes involved in angiogenesis and reprogramming energy metabolism [38,39].

HIF-1α, the best-characterized isoform, controls the expression of more than 100 genes, and in endothelial cells is estimated to affect more than 2% of the human genome [31,32]. Its half-life is only a few minutes [37], and expression occurs in many organs, such as the brain, heart, lungs, liver, kidney and pancreas [40]. Overexpression of HIF-1α has been observed in various types of cancer, where it has been shown to have prognostic significance in tumor progression [41,42]. Under normoxia, HIF-1α undergoes rapid proteasomal degradation, while under hypoxia it is stabilized and translocates to the cell nucleus, where it binds to HIF-1β to form an active transcriptional complex [43,44]. HIF-1 complexes bind to hypoxia-responsive elements (HREs) in the promoters of target genes, regulating adaptive processes including angiogenesis, metabolic reprogramming and cell survival [45].

HIF-1α stabilization plays a key role in rapidly proliferating, poorly vascularized solid tumors, in which the oxygen partial pressure can be <10 mmHg [46]. As little as three hours of exposure of tumor cells to hypoxia in vitro leads to stabilization of HIF-1α [47]. Under hypoxic conditions, HIF-1α affects tumor metabolism by promoting anaerobic glycolysis and inhibiting the TCA cycle, which increases the glucose dependence of tumor cells [48]. It induces the expression of glucose transporters (GLUT1, GLUT3, GLUT4) and glycolytic enzymes such as phosphofructokinase 1 (PFK1) [49], hexokinase 2 (HK2) [50] and lactate dehydrogenase A (LDHA) [51], promoting ATP production independent of mitochondrial activity [52,53].

Upregulation of LDHA by HIF-1α drives the conversion of pyruvate to lactate even in the presence of oxygen, a hallmark of the Warburg effect. The accumulation of lactate in the extracellular space lowers the pH, creating an acidic tumor microenvironment that favors cancer cell survival, invasiveness, and resistance to apoptosis. Acidification also suppresses local immune responses by impairing cytotoxic T-cell function and promoting the recruitment of immunosuppressive cells such as regulatory T-cells and myeloid-derived suppressor cells. Additionally, the acidic environment can activate proteases that degrade the extracellular matrix, facilitating tumor invasion and metastasis. Together, these processes link HIF-1α-driven metabolic reprogramming directly to aggressive tumor behavior and poor prognosis [54,55].

In addition, HIF-1α decreases pyruvate dehydrogenase (PDH) activity, reducing the conversion of pyruvate to acetyl-CoA, which affects lipid biosynthesis [56]. In response, tumor cells activate alternative metabolic pathways, including PPARγ-dependent fatty acid uptake [57] and isocitrate dehydrogenase (IDH)-mediated reversal of the TCA cycle [58]. HIF-1α also regulates glutamine metabolism through activation of glutaminase 1 (GLS1) [59] and inhibits mitochondrial biogenesis through induction of MXI1 expression [60], resulting in reduced oxygen consumption. These mechanisms contribute to increased survival and growth of cancer cells, making HIF-1α a key regulator of tumor metabolism [61]. Beyond its role in angiogenesis, HIF-1α also promotes a metabolic shift known as the Warburg effect, in which cancer cells preferentially rely on glycolysis rather than oxygen-dependent energy production, allowing them to survive in hypoxic environments.

HIF-1α also plays a fundamental role in tumor angiogenesis [62]. Stabilization and activation of this factor occur as a result of both hypoxia and genetic mutations. Activation of oncogenic signaling pathways, such as IGF, Ras and Src, can increase HIF-1α expression, as can inactivation of tumor suppressor genes, including pVHL, p53 and PTEN [63,64,65]. Once activated, HIF-1α forms a complex with the coactivators P300/CBP and HIF-1β, and then initiates the expression of pro-angiogenic factors, leading to the formation of new blood vessels that promote tumor growth and progression [66].

Additionally, HIF-1α plays a key role in tumor metastasis, mainly through induction of epithelial–mesenchymal transition (EMT) [67]. It interacts with TGF-β [68], enhancing its signaling through SMAD and non-SMAD pathways [69,70], and influences the activation of the Wnt/β-catenin pathway [71], which promotes invasiveness and metastasis. HIF-1α induces the expression of key EMT factors, such as TWIST [72,73], Snail [74,75], Slug [76,77], SIP1 [78] and ZEB1 [79], both through direct binding to their promoters and epigenetic mechanisms, such as HDAC3 recruitment [80]. In addition, HIF-1α regulates the expression of immunosuppressive molecules in tumor cells [81,82] and interacts with integrin kinase [83] and the FoxM1 pathway [84] to intensify EMT. HIF-1α activation also promotes invasion through induction of extracellular matrix metalloproteinases, leading to degradation of the basement membrane and facilitating the penetration of tumor cells into surrounding tissues [85,86]. In addition, HIF-1α controls the expression of lysyl oxidase [87], promoting the formation of a pre-metastatic niche, and regulates the expression of the MET tyrosine kinase receptor [88], enhancing tumor migration and invasiveness.

Measurement of serum HIF-1α levels has been evaluated for its diagnostic value in various cancers [89,90,91,92,93,94,95,96]. In the present study, we report the results of analyzing serum HIF-1α levels in patients with malignant salivary gland neoplasms. 

## 2. Materials and Methods

### 2.1. Serum Samples

The study was conducted at the Department of Otolaryngology, Pediatric Otolaryngology and Laryngologic Oncology of the University Clinical Hospital No. 1 in Rzeszow.

To determine the concentration of HIF-1a factor, use a serum separator tube and let the samples coagulate for two hours at room temperature before centrifuging for 20 min at about 1000× *g*. Serum samples were collected from 30 patients diagnosed with malignant salivary gland malignancies. The cohort consisted of 17 women and 13 men, with a mean age of 62.8 years (range 50–79 years). The study population included several histological subtypes, predominantly mucoepidermoid carcinoma, adenoid cystic carcinoma, acinic cell carcinoma with high-grade transformation, and salivary duct carcinoma.

### 2.2. Sample Preparation Procedure

Before direct use, all kit components and samples (serum) were equilibrated to room temperature (18–25 °C) and analyzed for HIF-1α by immunoenzymatic assay using kits with the catalog number W18Z26H96U (Cloud-Clone Corp. Katy, TX, USA).

### 2.3. Characteristics of Enzyme-Linked Immunoassay Test

The use of an immunoenzymatic assay using the W18Z26H96U kit enabled the determination of HIF-1α in in vitro samples. According to the manufacturer, the ELISA kits are tests with high sensitivity and excellent specificity for detecting HIF-1α.

### 2.4. HIF-1α Test Kit

Wells were defined on the plate for diluted standards (7 wells), blanks (1 well) and samples. Preparation of the test kit in each well was carried out in the following steps: (1) add 100 µL of standard, blank and sample, cover and incubate for 1 h at 37 °C; (2) remove liquid from each well, do not wash; (3) add 100 µL of Detection Reagent A and incubate for 1 h at 37 °C; (4) aspirate and wash 3 times with 350 µL Wash Solution and let stand for 1–2 min. Remove the remaining liquid completely from all wells by decantation; (5) add 100 µL of working solution of Detection Reagent B to each well, cover and incubate for 30 min at 37 °C; (6) aspirate and wash 5 times; (7) add 90 µL of substrate solution, cover and incubate in the dark for 10–20 min at 37 °C; (7) add 50 µL of stop solution, gently mix and remove any water droplets; (8) immediately take readings at 450 nm.

All chemical analyses were performed in triplicate. HIF-1α content was expressed as mean ± standard deviation.

Absorbance at 450 nm was measured using an ELISA plate reader (Infinite® 200 PRO, Tecan Group Ltd., Männedorf, Switzerland). A standard curve was generated by preparing 8 mixtures (0 pg/mL; 15.6 pg/mL, 31.2 pg/mL, 62.5 pg/mL, 125 pg/mL, 250 pg/mL, 500 pg/mL and 1000 pg/mL).

## 3. Results

### 3.1. Clinicopathological Characteristics of the Study Cohort

The clinicopathological characteristics of the study cohort are summarized in Table 1. The study included 30 patients with malignant salivary gland malignancies representing several histological subtypes, grades, stages, and anatomical locations. The cohort consisted of 17 women and 13 men, with a mean age of 62.8 years (range 50–79 years). A cohort of six healthy individuals was included as a control group to establish a normal physiological baseline for serum HIF-1α concentrations under standard normoxic conditions. Given the preliminary nature of this investigation and the relative rarity of malignant salivary gland neoplasms, this sample size was deemed sufficient to confirm the expected baseline absence of significant HIF-1α elevation, as HIF-1α remains highly unstable and rapidly degrades under non-hypoxic, healthy conditions. Although minor variations can occur due to intra-assay pipetting errors or baseline physiological fluctuations in healthy tissue, the massive difference between our healthy baseline (20 ± 5 pg/mL) and the oncology cohort (89.20 ± 44.56 pg/mL) vastly exceeds the typical 10–15% coefficient of variation (CV) characteristic of commercial ELISA systems. Therefore, any minor technical or biological error remains statistically negligible and does not obscure the clear demarcation between the normoxic baseline and tumor-induced hypoxia. standard commercial ELISA kit has an intra-assay error margin (CV) of around 5% to 10%. For a result of 20 pg/mL, a 10% error margin means the true value is between 18 pg/mL and 22 pg/mL. A baseline of 20 pg/mL sits safely above the typical minimum detectable dose (MDD) of modern commercial HIF-1α ELISA kits (which usually detect down to 5–10 pg/mL), proving your healthy control results are true signals, not background technical noise.

### 3.2. HIF-1α Levels in the Serum of Salivary Gland Cancer Patients

Individual serum HIF-1α concentrations are presented in Table 1. The mean serum HIF-1α concentration was 89.20 ± 44.56 pg/mL with a threshold of 48.93 pg/mL.

### 3.3. Exploratory Analysis of HIF-1α According to Clinicopathological Characteristics

An exploratory statistical analysis was performed to assess potential associations between serum HIF-1α levels and selected clinicopathological characteristics of the study cohort. The results are summarized in Table 2. Due to the limited sample size and the non-normal distribution of the data, non-parametric statistical tests were applied. Comparisons between stage II and stage III tumors were performed using the Mann–Whitney U test, whereas comparisons among histological subtypes and tumor grades were conducted using the Kruskal–Wallis test.

Patients with stage III tumors exhibited higher mean serum HIF-1α levels than patients with stage II disease (98.44 pg/mL vs. 67.63 pg/mL, respectively). Although this difference did not reach statistical significance, a strong trend toward higher HIF-1α concentrations in more advanced disease was observed (Mann–Whitney U test, U = 53.0, *p* = 0.064).

Similarly, serum HIF-1α concentrations tended to increase with increasing tumor grade. Mean HIF-1α levels were 69.25 pg/mL in grade II tumors, 79.95 pg/mL in intermediate-grade tumors, 76.89 pg/mL in acinic cell carcinomas with high-grade transformation, and 105.23 pg/mL in high-grade tumors. However, these differences were not statistically significant (Kruskal–Wallis test, H = 1.72, *p* = 0.633).

When analyzed according to histological subtype, salivary duct carcinoma demonstrated the highest mean serum HIF-1α concentration (125.23 pg/mL). Lower mean values were observed in mucoepidermoid carcinoma (82.79 pg/mL), acinic cell carcinoma with high-grade transformation (76.89 pg/mL), and adenoid cystic carcinoma (69.25 pg/mL). Despite these descriptive differences, no statistically significant differences were identified among histological subtypes (Kruskal–Wallis test, H = 2.90, *p* = 0.408).

Notably, three of the four highest individual HIF-1α concentrations were observed in patients with salivary duct carcinoma. However, given the small number of cases and the influence of individual high values, this observation should be interpreted with caution and cannot be considered evidence of increased activation of hypoxia-related signaling pathways in this histological subtype.

Overall, although no statistically significant associations were identified, the observed trends indicate that higher serum HIF-1α levels may be associated with more advanced disease stage, higher tumor grade, and aggressive histological subtypes. In particular, the near-significant association observed for tumor stage (*p* = 0.064) warrants further investigation in larger cohorts.

**Table 2:** Exploratory analysis of serum HIF-1α according to clinicopathological characteristics.

Variable	Category	*n*	Mean HIF-1α (pg/mL)	*p*-Value
Stage	Stage II	9	67.63	0.064
	Stage III	21	98.44	
Grade	Grade II	6	69.25	0.633
	Intermediate	6	79.95	
	High-grade transformation	4	76.89	
	High	14	105.23	
Histology	Adenoid cystic carcinoma	6	69.25	0.408
	Acinic cell carcinoma (HGT)	4	76.89	
	Mucoepidermoid carcinoma	13	82.79	
	Salivary duct carcinoma	7	125.23	

## 4. Discussion

In this study, we analyzed the serum levels of HIF-1α in salivary gland cancer patients. The mean concentration and standard deviation were 89.20 ± 44.56 pg/mL.

An exploratory analysis of clinicopathological characteristics revealed several potentially relevant trends. Patients with stage III tumors exhibited higher mean serum HIF-1α levels than those with stage II disease (98.44 pg/mL vs. 67.63 pg/mL). Similarly, higher-grade tumors tended to demonstrate increased HIF-1α concentrations, with mean values of 69.25 pg/mL in grade II tumors, 79.95 pg/mL in intermediate-grade tumors, and 105.23 pg/mL in high-grade tumors. When analyzed according to histological subtype, salivary duct carcinoma showed the highest mean serum HIF-1α concentration (125.23 pg/mL), whereas lower values were observed in adenoid cystic carcinoma (69.25 pg/mL), acinic cell carcinoma with high-grade transformation (76.89 pg/mL), and mucoepidermoid carcinoma (82.79 pg/mL). Although these observations should be interpreted with caution because of the limited sample size and histological heterogeneity of the cohort, they represent descriptive observations only and should not be interpreted as evidence of an association because of the limited sample size and histological heterogeneity of the cohort.

The detection of circulating HIF-1α in patients with salivary gland malignancies is biologically plausible and was not entirely unexpected, given previous reports demonstrating HIF-1α overexpression in salivary gland tumor tissues. However, the present study extends these observations by providing the first quantitative assessment of serum HIF-1α levels in this patient population and by exploring their potential association with clinicopathological characteristics.

Although no statistically significant differences were identified, descriptive trends toward higher serum HIF-1α concentrations in advanced-stage and high-grade tumors may warrant further investigation in larger cohorts. At present, these observations should be considered hypothesis-generating rather than evidence of a true biological association. Nevertheless, these descriptive trends are biologically plausible and consistent with the established role of HIF-1α as a central regulator of cellular adaptation to hypoxia [38,42,62]. As tumors progress, increasing metabolic demands and inadequate vascularization frequently result in the development of hypoxic regions, leading to HIF-1α stabilization and activation of downstream pathways involved in angiogenesis, metabolic reprogramming, invasion, and metastasis [46,62,67]. Although higher mean serum HIF-1α concentrations were observed in advanced-stage and high-grade tumors, these differences were not statistically significant. Therefore, any potential relationship with hypoxia-driven signaling remains speculative and requires confirmation in larger, adequately powered studies. Similarly, although salivary duct carcinoma exhibited the highest mean serum HIF-1α concentration in our cohort, this finding was based on a very small subgroup and was influenced by several high individual values. Therefore, it should be regarded solely as an exploratory observation requiring validation in larger cohorts [97,98,99,100,101,102,103,104,105,106,107,108,109,110,111].

Previous experimental studies have provided strong evidence that HIF-1α is actively involved in the progression of salivary gland malignancies rather than serving solely as a marker of hypoxia. In adenoid cystic carcinoma (ACC), elevated HIF-1α expression has been associated with increased invasive potential and metastatic behavior. Silencing of HIF-1α using siRNA significantly reduced tumor cell invasion, whereas pharmacological inhibition of HIF-1α using agents such as 17-AAG and echinomycin suppressed invasive activity and demonstrated anti-metastatic effects in experimental models. Furthermore, downregulation of HIF-1α in hypoxic ACC cells was shown to inhibit proliferation, migration, invasion, angiogenesis, and VEGF production while simultaneously promoting apoptosis. Reduced expression of matrix metalloproteinase-2 (MMP-2) and VEGF following HIF-1α inhibition further supports the central role of this transcription factor in regulating pathways associated with tumor progression and neovascularization. Collectively, these findings indicate that HIF-1α is not only a biomarker of tumor hypoxia but also a functional driver of aggressive tumor behavior in salivary gland cancers. The present study extends these observations by demonstrating measurable circulating HIF-1α levels in patients with salivary gland malignancies and by showing descriptive associations between higher serum HIF-1α concentrations, advanced tumor stage, higher histological grade, and aggressive histological subtypes. Although serum measurements cannot directly reflect intratumoral HIF-1α activity, the observed trends are consistent with previous tissue- and cell-based studies implicating HIF-1α in salivary gland cancer progression [112,113].

Recent evidence further supports the importance of hypoxia-related signaling in malignant salivary gland neoplasms. Cardoso et al. demonstrated that HIF-1α is deregulated in malignant salivary gland tumors, with increased expression associated with malignant transformation and tumor progression, highlighting its potential role as a biomarker of tumor hypoxia and biological aggressiveness [114]. More recently, a systematic review and meta-analysis by Maheswari et al. confirmed that overexpression of HIF-1α and HIF-2α is consistently associated with adenoid cystic carcinoma and contributes to tumor progression through the regulation of angiogenesis, epithelial–mesenchymal transition, invasion, and metastatic potential. These findings provide additional biological support for the investigation of circulating HIF-1α as a potential biomarker in patients with malignant salivary gland neoplasms [115].

This highly dynamic oxygen-dependent regulation may partly explain the substantial variability observed in serum HIF-1α concentrations in our study. Because HIF-1α has a short biological half-life and is sensitive to transient changes in tissue oxygenation, its circulating levels may reflect not only tumor burden but also differences in tumor vascularization, hypoxic microenvironment, and systemic metabolic status among patients. These biochemical characteristics should be considered when interpreting ELISA-based measurements, particularly in the absence of established reference values for salivary gland malignancies [110,116,117].

Although HIF-1α is predominantly an intracellular transcription factor with a biological half-life of only a few minutes under normoxic conditions, several mechanisms may explain its detectability in peripheral blood [38,42]. First, hypoxic tumor cells undergoing apoptosis, necrosis, or other forms of membrane disruption may release intracellular proteins, including HIF-1α, into the extracellular space following loss of membrane integrity [118]. Second, HIF-1α may be protected from immediate degradation by encapsulation within extracellular vesicles, particularly exosomes and apoptotic bodies, which are increasingly recognized as carriers of intracellular proteins released by hypoxic cancer cells. In addition, circulating HIF-1α may originate not only from tumor tissue but also from activated stromal, endothelial, or inflammatory cells exposed to hypoxic microenvironments [119]. Consequently, serum HIF-1α should not be interpreted as a direct measure of intracellular HIF signaling, but rather as an indirect surrogate reflecting the overall burden of hypoxia-associated cellular injury and turnover [120].

From an analytical perspective, several studies have demonstrated that circulating HIF-1α can be reproducibly quantified using commercially available sandwich ELISA assays, despite its intracellular localization. The measurable concentrations reported in both malignant and non-malignant diseases indicate that HIF-1α remains sufficiently stable after blood collection when standardized pre-analytical procedures, including rapid serum separation, appropriate storage conditions, and freeze–thaw minimization, are applied. Nevertheless, considerable variability exists among commercially available ELISA kits because of differences in antibody specificity, calibration standards, detection ranges, and recognition of free versus vesicle-associated HIF-1α. Therefore, absolute concentrations obtained in different studies are not directly comparable, emphasizing the importance of standardized analytical protocols and validation within individual laboratories. In the present study, all samples were processed using identical pre-analytical procedures and analyzed with the same commercially available ELISA kit in triplicate, thereby minimizing analytical variability within the study cohort, although inter-study comparisons should still be interpreted with caution [121,122].

An additional limitation is that HIF-1α is primarily an intracellular transcription factor localized in the cytoplasm and nucleus rather than a constitutively secreted circulating protein. Under physiological conditions, its extracellular presence is minimal because HIF-1α undergoes rapid oxygen-dependent degradation through the prolyl hydroxylase–von Hippel–Lindau pathway, resulting in a very short half-life. Therefore, measurable serum concentrations most likely reflect secondary release from tumor cells undergoing hypoxic stress, apoptosis, necrosis, or membrane disruption. Furthermore, hypoxic tumors may release HIF-1α through extracellular vesicles, including exosomes and apoptotic bodies, which can protect intracellular proteins from immediate degradation and contribute to their detectability in peripheral blood. As a result, circulating HIF-1α levels may depend not only on tumor burden itself, but also on the degree of intratumoral hypoxia, cellular turnover, extracellular vesicle production, and pre-analytical factors related to sample handling, all of which may contribute to the substantial interindividual variability observed in serum measurements [117,123,124].

Given the important role of HIF-1α in the pathogenesis of salivary gland malignancies, its potential as a predictive factor, which likely depends on the type of cancer, has been analyzed. HIF-1α expression has been shown to correlate with TNM stage and histologic differentiation grade of adenocarcinoma [107]. In contrast, in salivary duct carcinoma (SDC), where HIF-1α was evaluated as one of the biomarkers of hypoxia, no significant effect on locoregional control, risk of distant metastasis or overall survival was found [125].

To the best of our knowledge, the present study is the first to measure serum HIF-1α levels in patients with malignant salivary gland neoplasms. The mean serum HIF-1α concentration observed in our cohort (89.20 ± 44.56 pg/mL) was higher than that reported in several studies involving healthy individuals or patients with non-malignant diseases, in which mean values ranged from 0.23 to 37.21 pg/mL. However, substantially higher concentrations, reaching 1410 pg/mL and even 9200 pg/mL, have also been described in other non-malignant clinical settings. These marked differences most likely reflect heterogeneity in study populations, underlying disease processes, pre-analytical conditions, and ELISA methodologies. Consequently, the lack of standardized reference values complicates the interpretation of serum HIF-1α concentrations and limits direct comparisons between studies.

Evaluation of 20 non-cancer subjects showed a baseline serum HIF-1α concentration of 22.99 ± 9.10 pg/mL [93] and 23.84 ± 8.15 pg/mL [95]. An ELISA evaluation of 40 non-cancer patients demonstrated a mean serum HIF-1α concentration of 1410 ± 320 pg/mL [89], 123 pg/mL [92] and 37.21 ± 4.33 pg/mL [94]. In contrast, analysis of a cohort of 46 non-cancer controls yielded a significantly higher mean serum HIF-1α level of 9200 ± 3400 pg/mL [91]. A separate study involving 101 non-oncological individuals reported a much lower baseline concentration of 0.23 ± 0.11 pg/mL [90]. These striking discrepancies in baseline serum HIF-1α concentrations among non-cancer cohorts can be explained by several methodological and biological factors. First, HIF-1α is highly sensitive to pre-analytical sample handling; any delay in blood processing or variation in centrifugation speed can cause ex vivo hypoxia in the collection tube, leading to artificial stabilization and release of HIF-1α by peripheral blood cells. Second, the commercial ELISA kits used across these studies lack international standardization, featuring different capture antibodies, detection limits, and standard curve calibrations. Finally, the clinical characteristics of ‘non-cancer’ control groups often varied, potentially including patients with underlying inflammatory, metabolic, or cardiovascular conditions that physiologically upregulate systemic HIF-1α despite the absence of a malignancy.

The mean serum HIF-1α concentration observed in the present study was 89.20 ± 44.56 pg/mL. Consequently, physiological serum HIF-1α levels in healthy individuals remained strictly below the threshold of 48.93 pg/mL and ranged between 20.00 and 40.00 pg/mL due to continuous normoxic protein degradation, which corresponds to the statistical distribution and aligns with baseline-concordant literature [93,94,95].

Comparison with previously published studies demonstrates that circulating HIF-1α concentrations vary substantially among different malignancies. The mean serum HIF-1α concentration observed in patients with malignant salivary gland neoplasms was higher than that reported for non-small cell lung cancer (50.66–63.10 pg/mL) [94,96] but lower than the concentrations reported for primary liver cancer (154.94–160.61 pg/mL) [93,95] and urothelial carcinoma of the bladder (345 pg/mL) [92]. Markedly higher HIF-1α levels have been described in hepatocellular carcinoma (1901.62 pg/mL) [94], meningioma (2300 ± 670 pg/mL) [89], glioblastoma (2610 ± 1940 pg/mL) [89], gastric cancer (75,400 ± 14,700 pg/mL) [91], colorectal adenocarcinoma [90] and colorectal adenoma [90]. These substantial differences most likely reflect variations in tumor biology, the degree of intratumoral hypoxia, tumor burden, vascularization, disease stage, and analytical methodology, including differences in patient selection, sample processing, and ELISA assays. Consequently, although the serum HIF-1α concentration observed in our cohort falls within the wide spectrum reported for solid malignancies, direct comparisons between studies should be interpreted with caution because of the lack of standardized analytical protocols and validated reference ranges.

Overall, these findings suggest that serum HIF-1α concentrations in malignant salivary gland neoplasms are within the range reported for solid malignancies but are characterized by considerable inter-study variability.

Our findings suggest that serum HIF-1α may represent a promising biomarker associated with malignant salivary gland neoplasms. Nevertheless, the diagnostic performance of serum HIF-1α cannot be reliably assessed in the present study because of the limited number of healthy controls (*n* = 6) and the absence of patients with benign salivary gland neoplasms. Therefore, these findings should be considered preliminary and require validation in larger, well-characterized cohorts including both healthy individuals and patients with benign salivary gland tumors.

Several important limitations should be acknowledged. First, the observed HIF-1α concentrations demonstrated considerable interindividual variability (89.20 ± 44.56 pg/mL), which is consistent with previous reports describing substantial variation in circulating HIF-1α levels. Such variability complicates the establishment of precise diagnostic thresholds and limits immediate clinical applicability. Second, as this is the first study evaluating serum HIF-1α levels in patients with salivary gland malignancies, validated reference values are currently unavailable, restricting comprehensive interpretation of the obtained results. Third, the study cohort was relatively small and included multiple histological subtypes with distinct biological characteristics, limiting the statistical power of subgroup analyses. Consequently, the exploratory associations observed between serum HIF-1α levels and clinicopathological variables should be interpreted cautiously. Finally, tissue expression of HIF-1α was not assessed in the present cohort, preventing direct comparison between circulating and intratumoral HIF-1α levels, while the absence of longitudinal follow-up data precluded evaluation of the prognostic significance of serum HIF-1α concentrations.

In summary, this study represents the first evaluation of serum HIF-1α levels in patients with salivary gland malignancies. The mean serum HIF-1α concentration was 89.20 ± 44.56 pg/mL, although substantial interindividual variability was observed. Exploratory analyses identified descriptive differences in serum HIF-1α concentrations across tumor stage, grade, and histological subtype. However, because of the limited sample size, these findings should be considered hypothesis-generating and require confirmation in larger studies. Our findings suggest that circulating HIF-1α may represent a promising biomarker associated with malignant salivary gland neoplasms and their biological aggressiveness. However, because the present study did not include patients with benign/malignant salivary gland neoplasms or healthy controls, its diagnostic value cannot be established. Future studies, including appropriate control groups, are warranted to determine the diagnostic and prognostic utility of serum HIF-1α.

## Figures and Tables

**Table 1:** Clinicopathological characteristics of the study cohort.

Sample	Sex	Age	Histology	Grade	Stage	Location	HIF-1α Concentration [pg/mL]
1	W	65	Mucoepidermoid carcinoma	High	III	Parotid	76.14
2	W	68	Adenoid cystic carcinoma	II	II	Submandibular	66.21
3	W	72	Acinic cell carcinoma	High-grade transformation	III	Parotid	99.96
4	M	61	Mucoepidermoid carcinoma	Intermediate	III	Parotid	66.64
5	W	64	Acinic cell carcinoma	High-grade transformation	II	Parotid	69.21
6	M	66	Mucoepidermoid carcinoma	High	III	Parotid	58.32
7	M	70	Salivary duct carcinoma	High	III	Parotid	105.21
8	M	61	Salivary duct carcinoma	High	III	Parotid	75.36
9	M	78	Salivary duct carcinoma	High	II	Parotid	58.93
10	M	50	Adenoid cystic carcinoma	II	II	Submandibular	63.79
11	W	67	Salivary duct carcinoma	High	III	Parotid	248.25
12	M	79	Salivary duct carcinoma	High	II	Parotid	65.07
13	M	61	Mucoepidermoid carcinoma	Intermediate	III	Parotid	113.82
14	W	59	Mucoepidermoid carcinoma	Intermediate	III	Parotid	63.96
15	W	65	Acinic cell carcinoma	High-grade transformation	III	Parotid	60.39
16	M	62	Salivary duct carcinoma	High	III	Parotid	148.00
17	W	58	Adenoid cystic carcinoma	II	II	Submandibular	92.21
18	M	56	Mucoepidermoid carcinoma	Intermediate	III	Parotid	78.68
19	W	60	Mucoepidermoid carcinoma	High	III	Parotid	76.82
20	W	67	Mucoepidermoid carcinoma	High	III	Parotid	180.21
21	W	55	Adenoid cystic carcinoma	II	II	Submandibular	78.29
22	W	59	Acinic cell carcinoma	High-grade transformation	III	Parotid	78.00
23	W	63	Adenoid cystic carcinoma	II	II	Submandibular	66.04
24	M	60	Mucoepidermoid carcinoma	Intermediate	III	Parotid	102.82
25	W	65	Mucoepidermoid carcinoma	Intermediate	III	Parotid	53.75
26	W	59	Mucoepidermoid carcinoma	High	III	Parotid	80.11
27	M	53	Mucoepidermoid carcinoma	High	III	Parotid	68.82
28	M	56	Mucoepidermoid carcinoma	High	III	Parotid	56.21
29	M	62	Salivary duct carcinoma	High	III	Parotid	175.82
30	W	60	Adenoid cystic carcinoma	II	II	Submandibular	48.93

## Data Availability

The original contributions presented in this study are included in the article. Further inquiries can be directed to the corresponding author.
